# Clinical characteristics and gaps in palliative care among tracheostomized children: a retrospective observational study^☆^

**DOI:** 10.1016/j.jped.2025.101480

**Published:** 2025-12-12

**Authors:** Luziane Lais Sabino Silva Luna, Sheyla Suelle dos Santos Levy, Luciana Santana Lima, Maria do Carmo Menezes Bezerra Duarte

**Affiliations:** Instituto de Medicina Integral Prof. Fernando Figueira - IMIP, Recife, PE, Brazil

**Keywords:** Tracheostomy, Palliative care, Children

## Abstract

**Objective:**

To describe the clinical characteristics of tracheostomized children in Northeastern Brazil and to identify existing gaps in the indication and implementation of palliative care.

**Methods:**

This retrospective descriptive study reviewed medical records of children under 15 years who underwent tracheostomy between 2008 and 2019 at a quaternary referral center.

**Results:**

Sixty-five tracheostomized patients with indications for palliative care were analyzed. The main reasons for palliative classification were acute life-threatening conditions (50.7 %) and chronic life-threatening conditions (20 %). The most frequent tracheostomy indications were prolonged invasive mechanical ventilation (46.1 %) and severe upper airway obstruction (23 %). The mortality rate was 30.7 %, higher among infants, severely malnourished children, those with Lansky scores below 40 % before tracheostomy, congenital heart disease, bronchopulmonary dysplasia, and multiple comorbidities. Only 10.8 % received palliative care consultations during hospitalization, all in the end-of-life care phase.

**Conclusions:**

The results highlight a critical gap in the provision of palliative care to tracheostomized children. Early involvement of a multidisciplinary palliative care team is crucial to support clinical decision-making, family-centered care, and ensure quality of life. The proposed framework may facilitate timely referral and airway planning in the Intensive therapy, although further validation and qualitative research in various healthcare settings are needed.

## Introduction

Tracheostomy is increasingly performed electively in critically ill children and infants under one year of age. It was first performed in the treatment of diphtheria in the 19th century, and its indication in infants and children is summarized in five main categories: airway immaturity, congenital obstructive anomalies of the upper airways (UWA), acquired UWA obstructions, head and neck tumors, and trauma. In recent decades, it has been mostly used to treat anatomical UWA obstructions and the need for prolonged invasive mechanical ventilation.^1^, ^2^, ^3^, ^4^, ^5^

There is still no consensus regarding the optimal timing for tracheostomy in patients with complex clinical conditions (CCC). Like other forms of respiratory support, tracheostomy may serve as a bridge to the recovery of pulmonary function or as a destination therapy, requiring careful consideration of the implications of chronic invasive mechanical ventilation (IMV).^6^

For patients with CCC and life-limiting illnesses, the concept of palliative tracheostomy emerged with the aim of promoting respiratory comfort, ensuring the child's safe mobility, and reducing ventilatory support parameters to a minimum. This increases the patient's waking hours and interaction with the environment and family, facilitating parental involvement in care and enabling discharge from the intensive care unit (ICU) to a more appropriate environment, such as pediatric wards or hospices.^7^

The 2022 mapping of pediatric palliative care (PC) in Brazil revealed an increase in these services over the past decade, demonstrating recent developments in the field. At the same time, the distribution of these services is uneven across Brazilian regions, and the availability of beds or hospices is minimal. Of the beds in PC services, only 13 % are dedicated to pediatric PC.^8^ The pediatric PC service at IMIP began in 2016 with intensivists who started applying and disseminating their knowledge in pediatric intensive care units. The adult PC service already existed, and this team was consulted in case of doubts.

Through open dialogue involving the patient, family, and a multidisciplinary team, the decision to perform a tracheostomy can be a valuable component in formulating a comprehensive care plan.^9^

This study aimed to describe the clinical characteristics of tracheostomized children in Northeast Brazil and to identify existing gaps in the indication and implementation of palliative care.

## Methods

This is a retrospective observational study using data from medical records, including children and adolescents under 15 years of age who underwent tracheostomy and were admitted to the pediatric ICU and inpatient wards of a quaternary teaching hospital in northeastern Brazil between 2008 and 2019. The period chosen was 10 years before starting discussions about CP with a multidisciplinary team. Patients who underwent emergency tracheostomy and those who had the procedure performed in another hospital prior to transfer to the Instituto de Medicina Integral Prof. Fernando Figueira (IMIP) were excluded. IMIP is a non-profit philanthropic hospital that exclusively serves patients from the Brazilian Unified Health System (SUS) and includes a Center for Craniofacial Anomalies (CADEFI).

Medical records were identified using the hospital's electronic health record system, based on the Unified Supplementary Health Table (TUSS) procedure code for tracheostomy – 040401037-7 – for the period from 2008 to 2018. For the year 2019, records were identified through a manual review of surgical block logs for pediatric procedures. Subsequently, the medical records were reviewed at the Medical Records and Statistics Department using a specific data collection form.

The variables assessed included biological and clinical data, family involvement, palliative care, and discharge outcomes. Prolonged IMV was defined as mechanical ventilation for at least 30 consecutive days. Nutritional status was calculated using the weight-for-age percentile (% W/A) and BMI-for-age (% BMI/A), considering weight, age, sex, and height, and plotted on the World Health Organization (WHO) growth charts.^10^

Functionality was retrospectively assessed using the Palliative Performance Scale (PPS) – Lansky modified version for children under 16, based on data recorded in the medical charts. This scale is employed to assess the functional status of children and adolescents based on their level of play activity, mobility, social engagement, and autonomy, providing an overall overview of clinical condition through a decile-based scoring system ranging from 100 to zero.^11^ Performance was classified as: High (100–80 %), Moderate (70–50 %), Low (40–20 %), and Critical (10–0 %). A PPS-Lansky score below 40 % was considered indicative of eligibility for proportional palliative care (decisions that provide meaningful benefit and avoid non-beneficial or futile treatments).^11^

This research project was approved by the Research Ethics Committee of the IMIP (number 3.312.444). A waiver of the Informed Consent Form was requested due to the research being conducted using secondary data and the difficulty in locating the patients' guardians.

Data descriptive analysis was performed using absolute and relative frequencies for categorical variables and through central tendency and variability measures for numerical variables.

## Results

A total of 86 medical records with the tracheostomy procedure code were evaluated. Twenty-one patients were excluded: nine underwent the procedure in another hospital, seven did not undergo tracheostomy (coding error), three underwent emergency tracheostomy, and two had no procedure description in the medical chart. Therefore, 65 patients were included in the study.

Biological and clinical characteristics are presented in Table 1. The median age was three months, ranging from six days to 15 years, with 75.3 % of the children being six months old or younger. Nutritional status was assessed in 46 children (70.8 %), of whom 26.1 % were classified as severely malnourished. Comorbidities were present in 52 % of patients, the most common being congenital heart disease (21.5 %) and pulmonary conditions (12.3 %), particularly bronchopulmonary dysplasia.

**Table 1:** Characteristics of 65 children at the time of tracheostomy from 2008 to 2019.

Age	N	%
Newborns	7	10,7
1 month to 6 months	42	64,6
7 to 24 months	5	7
> 2 years	11	16,9
**Gender^1^ female**	32	50,7
%W/A (0–10 years) or %BMI/A (> 10 years)		
≥ 0	3/65	4,6
−2 a −3	6/65	9,2
< −3	17/65	26,1
Missing data	20/65	30
**No comorbidities**	31	47,7
**Comorbidities (type)**	34	52,3
Congenital heart disease	14	21,5
Lung diseases	8	12,3
CNS Diseases	6	9
Genetic diseases	6	9
**Comorbidities (numbers)**		
One comorbidity	24	36,9
Two comorbidities	8	12,3
> 2 comorbidities	2	3
**Indications for palliative care**	65	100 %
**I** – Children with acute, life-threatening conditions, where recovery may or may not be possible	33	50,7
**II** - Children with chronic, life-threatening conditions that can be cured or controlled for a long period, but that can cause death	13	20
**III** – Children with progressive, life-threatening conditions for whom no curative treatment is available	8	12,3
**IV-** Children with severe neurological conditions, not progressive, but which can cause deterioration and death	11	16,9
**V**- Newborns who are severely premature or have severe congenital anomalies.	0	—

N, number; 1, Some variables were not recorded in the medical records: for sex - 64 records; %W/A, weight/age percentile; %BMI/A, body mass index percentile/age; CNS, Central Nervous System.

The most frequent reasons for hospitalization were craniofacial anomalies (23 %), notably the Pierre Robin sequence, UWA obstructive disorders (20 %), congenital heart disease, and neurological disorders (13.8 % each). The primary indications for tracheostomy were prolonged IMV (46.1 %), severe UWA obstruction (23 %), and extubation failure (15.3 %). Among patients who underwent tracheostomy as a consequence of prolonged IMV, the primary underlying conditions included congenital heart disease (34.7 %), neurological disorders (26 %), prematurity associated with bronchopulmonary dysplasia (17 %), infectious diseases (13 %), and thoracic cystic hygroma and achondroplasia (4 % each).

Patient classification based on PC eligibility criteria is shown in Table 1. The main indications were acute life-threatening conditions (50.7 %) and chronic life-limiting conditions (20 %). The PPS-Lansky scores at admission and discharge were available for 51 patients in this study, and 76.4 % had scores below 40 % at the time of tracheostomy. Discussions between the intensive care pediatrician and family members about pediatric PC began in 2015 at the IMIP. Among all medical records analyzed in this study, only 10.8 % documented the content of discussions with family members and staff detailing a record care plan (review of clinical conditions and discussion with the family and patient to understand their values and desires, thus aligning expectations and goals of care). These discussions took place in the ICU and were led by the head of the pediatric ICU. In three cases, the conversation occurred after tracheostomy. Among the seven patients evaluated in this context, five (71 %) died during the same hospitalization (Table 2).

**Table 2:** Description of the seven patients approached by the palliative care team.

N*	Age (m)^1^	Underlying disease	Comorbidities	LOS	TRAC Indication	PC (before or after TRAC)	PPS –Lansky admittance	PPS –Lansky on the rise / death	Denouement
20	1	Cystic fibrosis	Genetic SD2 CHD Bone dysplasia	49	IMV prolonged	After	10	0	Death
27	22	CNS tumor	-	65	IMV prolonged Management of secretions	After	-	0	Death
38	4	CHD	Serious bronco-dysplastic	162	IMV prolonged	2 days before	10	0	Death
40	17	Complex CHD (ventricle unique)	Chronic hypoxia – neurological damage	78	IMV prolonged Secretion management	After	10	0	Death
49	2	CHD	Genetic SD (Down)	78	IMV prolonged	11 days	10	-	Hospital discharge
53	0,36	Tracheal membrane	CHD	55	Severe UAW obstruction	1 day before	-	0	Death
61	144	Infectious disease	-	88	IMV prolonged	12 days before	10	40	Hospital discharge

* N, number of the patient in the study; 1 - months; 2 – SD, Syndrom; LOS, lenght of hospital stay; TRAC, tracheostomy; PC, Palliative care; PPS, Performance personal scale; CNS, central nervous system; CHD, Congenital Heart Disease; IMV, invasive mechanical ventilation; UAW, upper airways.

Tracheostomy complications occurred in 12 (18.4 %) patients, nine of whom were under two years of age, and three were neonates. Among these, 75 % had one or more comorbidities. The most frequent complications were tracheostomy tube blockage due to secretion plugs (33.3 %) and accidental decannulation (25 %). One death (1.5 %) occurred due to a tuve obstruction. No patient was decannulated during the hospitalization in which the tracheostomy was performed. After the procedure, 20 % of the patients did not require further IMV; of these, 84.6 % had diseases UAW or abnormalities of craniofacial disorders. Forty-one patients (63 %) were weaned from IMV after tracheostomy, with a mean of 8.5 days (range: 0–48 days) and a median of two days.

Regarding hospital length of stay (LOS), the median was 65 days (range: 1–863 days), and 28 (43 %) patients remained hospitalized for more than 30 days after the tracheostomy. Overall, in-hospital mortality was 30.7 %, with a median age at death of 2.5 months; among these, 75 % had congenital heart disease, 41.6 % were severely malnourished, 30 % had bronchopulmonary dysplasia, and 5 % had craniofacial anomalies (Table 3). The median LOS in this group was 106.5 days. Among the 20 deceased patients, 17 had a recorded Lansky score, and all scored 40 % or less (end-of-life care) prior to the tracheostomy. The characteristics of patients according to the WHO PC classification are presented in Table 3.

**Table 3:** Clinical characteristics of tracheostomized patients who died according to WHO palliative care criteria.

PC indication WHO^a^ (N)	Age (months)	Underlying disease (N)	Comorbidities (N)	%W/A or BMI^b^ (N)	Lansky pré trach (N)	IMV Time after Trach (days)	LOS (days)
I (7)	0,2- 4	CHD - 4 Duodenal atresia – 1 Cervical Hygroma - 1 Arboviral disease – 1	0 - 2 1 - 4 2 - 1	0< %w/*i*<−2 - 3 −2< %p/*i* <−3 - 1 <−3 – 1 2< %p/*i* < 0 –1 IGN - 1	10 - 4 30 – 1 IGN - 2	0–29 IMV-D - 3 IGN - 1	37 - 188
II (5)	1–4	Cystic fibrosis - 1 Prematurity - 1 Choanal atresia - 1 Achondroplasia - 1 CHD - 1	0 - 2 1 - 1 2 - 1 3 - 1	<−3 - 2 −3 - 1 IGN - 2	10 - 2 20 – 2 30 - 1	8 - 1 IMV-D - 3 IGN - 1	49 - 863
III (2)	1 - 22	Trisomy of chromosome 18 - 1 CNS Tumor - 1	0 - 1 1 - 1	−3 - 1 IGN - 1	40 - 1 IGN - 1	5 - 1 IMV-D - 1	63 - 65
IV (6)	1 – 17	CHD - 5 MMC - 1	0 - 1 1 - 4 3 - 1	<−3 - 5 IGN - 1	10 - 6	0 - 16 IMV-D - 2 IGN - 1	78 - 219

a WHO, World Health Organization.

b %W/A, %weight/age and BMI, body mass index (WHO); PC, palliative care; N, number of patients; LOS, lenght of hospital stay; IMV, invasive mechanical ventilation; CHD, Congenital Heart Disease; IMV-D, invasive mechanical ventilation dependent; CNS, central nervous system; MMC, meningomyelocele congenital; IGN, ignored. Age and LOS were presented as minimum and maximum values.

## Discussion

This study describes the clinical characteristics and identifies gaps in the indication and implementation of PC among 65 tracheostomized children and adolescents hospitalized in a quaternary teaching hospital in northeastern Brazil from 2008 to 2019. IMIP exclusively serves patients covered by the Brazilian Unified Health System (SUS), which may limit the generalizability of these findings to settings with different sociocultural and healthcare structures. Approximately two-thirds of patients were younger than six months, and half presented with craniofacial anomalies or upper airway (UAW) defects and comorbidities, most frequently congenital heart disease. Tracheostomy was indicated in two-thirds of cases for prolonged IMV or UAW obstruction. All patients met WHO criteria for PC, with 50 % having acute life-threatening illness. The overall mortality rate was 30.7 %, and only 10.7 % were evaluated by a PC provider.

Understanding the clinical profile of tracheostomized pediatric patients supports healthcare planning and cost management of preventive and therapeutic interventions. Partially consistent with the present results, a study from Rio Grande do Sul involving 123 tracheostomized children reported that 56 % were younger than one year and 84 % had comorbidities, mainly neurological disorders (41 %).^12^ Similarly, a multicenter American study with 917 children showed that 48 % were six months or younger, with chronic lung disease (56 %), neurological impairment (48 %), and UAW anomalies (47 %) as the most prevalent comorbidities.^2^ This data aligns with these studies regarding patient age and comorbidity prevalence, but differ in the predominance of airway and cardiac disorders rather than neurological impairment, reflecting the local epidemiological profile. Variations in patient characteristics likely reflect hospital specialization; centers focused on trauma and neurosurgery tend to perform tracheostomy more often due to prolonged IMV or early indication following neurological injury.^13^

The lethality rates observed in this study (30.7 %) and those specifically associated with tracheostomy (1.5 %) were similar to figures reported in the literature, which range from 30–40 % and 0–8 %, respectively.^3^^,^^4^^,^^14^^,^^15^ Furthermore, as previously documented, higher mortality rates were seen among children with cardiac and neurological diseases compared to those with craniofacial anomalies or isolated airway obstruction.^2^ In a study by Schweiger et al. in southern Brazil, which evaluated tracheostomy complications, the mortality rate was 32 % among children with tracheostomies. These deaths were related to underlying comorbidities, primarily due to the large number of critically ill patients with chronic neurological conditions, and not directly to the presence of a tracheostomy.^12^

Malnutrition is a known risk factor for increased mortality in tracheostomized children and is associated with longer IMV and ICU stays. In this study, one-quarter of patients had severe malnutrition, underscoring their social vulnerability. Pernambuco, where IMIP is located, accounts for 18.7 % of Brazil’s population living in extreme poverty.^16^

In studies involving children with congenital heart disease, the involvement of the CP was associated with reduced use of mechanical circulatory support, ventilation, inotropes, or cardiopulmonary resuscitation at the end of life. This led to greater chances of children being awake and receiving enteral nutrition on the day of death, better advance care planning, lower hospital costs during the last seven days of life, and fewer invasive interventions at the end of life.^17^^,^^18^

In this case series, despite current recommendations, few patients were referred to PC teams. When assessments were made, they typically occurred late in the disease trajectory, were conducted by individual trained physicians rather than multidisciplinary teams, and often took place after invasive procedures (e.g., tracheostomy) or at the end of life. This pattern may reflect the misconception that PC begins only when curative treatment is no longer feasible — which is incorrect.

Palliative care should prioritize humanizing the relationship between the healthcare team, the patient, and their family, offering a compassionate response from diagnosis through end-of-life. Achieving this requires a multidisciplinary team including physicians, nurses, physiotherapists, psychologists, social workers, and other professionals. In the context of surgical palliative care, the primary aims of invasive interventions are to provide sustained symptom relief, restore organ function, enhance quality of life and body image, and optimize overall patient management. These benefits must be weighed judiciously against disease burden, functional status, length of hospitalization, surgical morbidity and mortality, and the potential need for further palliative interventions.^19^

Multidisciplinary assessment of the patient's mental status allows for early involvement in difficult conversations with the patient and their family, facilitates therapeutic planning, and enables informed decisions about the potential benefits or futility of prolonged mechanical ventilation and invasive procedures.^15^ Identifying ICU patients who would benefit from early assessment of the patient's mental status is relevant and essential.

In this study, the authors classified according to the WHO and calculated the PPS-Lansky scale based on medical record data to estimate the population of tracheostomized children who would be indicated for CP. The authors observed that everyone in the sample was indicated for one criterion or another. Decisions to initiate or intensify PC should be based on comprehensive assessments, considering clinical conditions, the child’s individual needs, and family preferences, rather than solely relying on specific PPS-Lansky scale thresholds. PC is indicated from the time of diagnosis of complex chronic conditions or in the setting of life-threatening acute illness.^20^ Notably low PPS-Lansky scores at admission (< 40 %) indicated that end-of-life care was appropriate. For most of these patients, hospitalizations were prolonged, and the outcomes were unfavorable. Functional scores aid in these assessments. Studies in the US have also shown that referrals for palliative care often occurred late in the course of the disease, with consultations occurring a median of 42 days before death in 31 % of children and 60 days before death in only 3.6 % of cases.^17^^,^^18^^,^^20^

Prolonged IMV and UAW obstruction were the primary indications for tracheostomy in this study, consistent with findings from high-income countries. This reflects the global trend of increasing availability of IMV for respiratory failure management.^4^^,^^15^^,^^21^

Although there is no consensus on the ideal timing for pediatric tracheostomy, systematic reviews and randomized clinical trials have categorized them as early or late. A meta-analysis revealed that early tracheostomy (within 14 days of intubation) significantly reduced hospital and ICU length of stay, although it had no impact on mortality. Other studies suggest that the timing of tracheostomy may depend more on the underlying disease than the duration of IMV.^2^^,^^15^^,^^22^, ^23^, ^24^

In this study, even after 30 days of tracheostomy, a significant proportion of patients (43 %) remained hospitalized. None were decannulated before discharge, likely due to contraindications to decannulation: limited access to airway endoscopy (AE) or inadequate control of the underlying disease. At IMIP, prolonged hospitalization may be related to challenges in accessing home care through SUS. Discharge requires the patient to be free of oxygen dependence, have home adaptations for tracheostomy care, and have trained caregivers. Additionally, disease management and associated comorbidities necessitate continuous care beyond airway and long-term ventilation needs.

According to the Brazilian National Academy of Palliative Care (ANCP), decision-making in PC must distinguish futile from potentially inappropriate interventions. The former offers no chance of achieving physiological goals, while the latter involves technical and individual value judgments that should be revisited and discussed prior to final decisions.^25^

Clinical decisions about tracheostomy in palliative settings should be made by multidisciplinary teams, working with caregivers, with caregivers playing a central role.^3^^,^^6^ A prospective study in the US assessed decisional conflict and regret among parents of tracheostomized children, reporting increased regret and decreased quality of life in the short term after tracheostomy. Feeling uninformed and pressured to decide emerged as the main sources of parental distress and regret.^6^

In spite of the relevance of this study, some limitations should be highlighted. Firstly, as this is a single-center study in a population living in low socioeconomic conditions, it may not reflect the results of other populations around the world. Secondly, due to the retrospective design and the fact that the hospital does not have electronic medical records, some medical records were not found in the hospital's physical archive, and others were incomplete. Thirdly, it was not possible to collect some patients' variables, such as weight, height, date of withdrawal from IMV after tracheostomy.

Despite these limitations, the study reinforces the need for early, multidisciplinary PC assessment for all pediatric ICU admissions — planned or unplanned — to enable personalized therapeutic planning, including airway management, tailored to the underlying disease, comorbidities, functionality, family expectations, and conditions.

**Figure 1:**
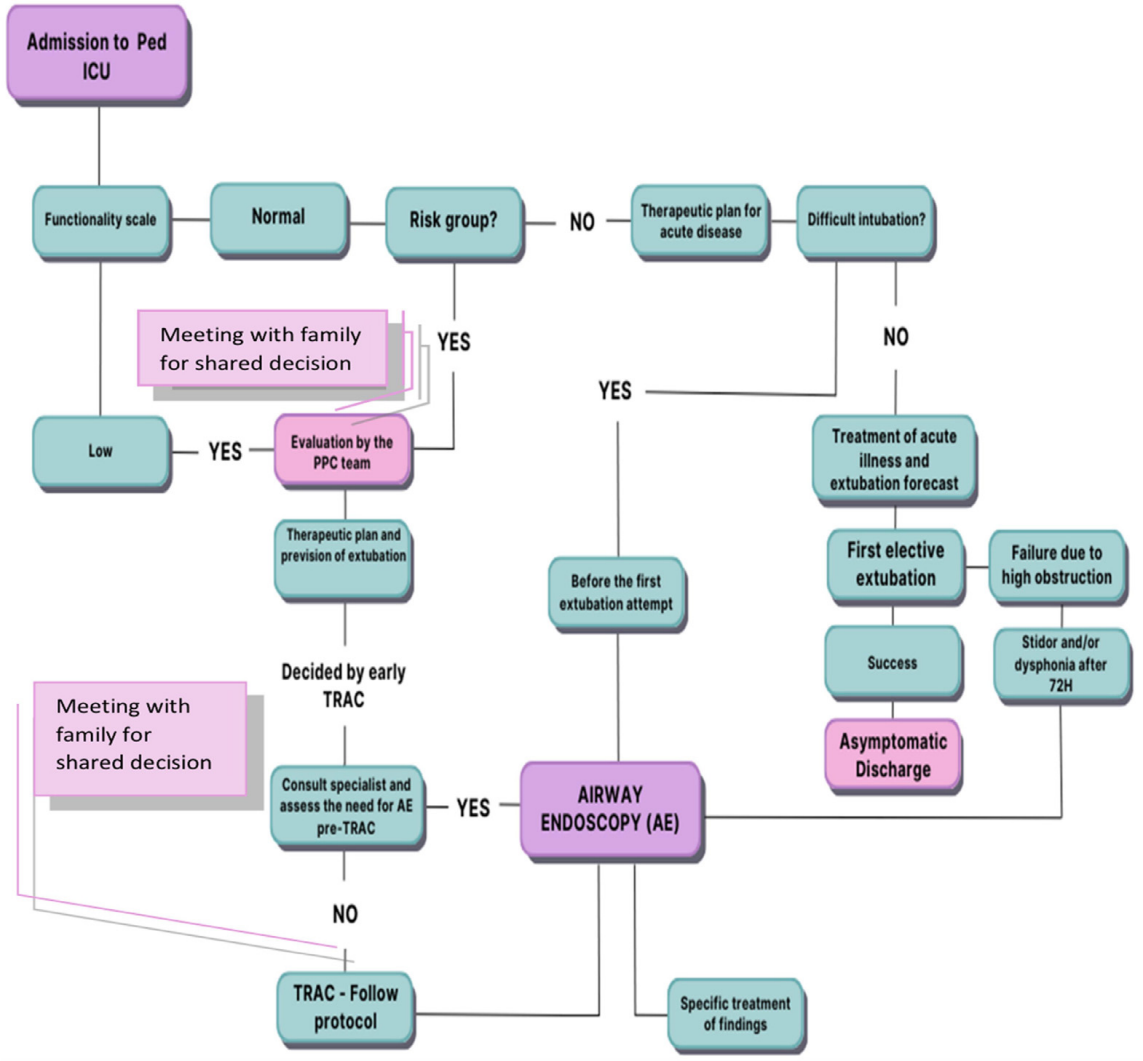
Framework to support clinical decision-making, identify indications for pediatric palliative care, and guide airway management in ICU patients.

Source: Authors’ own elaboration (2025). TRAC – tracheostomy; pediatric palliative care (PPC); Pediatric Intensive Care Unit (ICU). Risk group: Neonates with congenital or genetic disease, Infants under one year of age, Malnourished, Neurological diseases, Low functionality score on, admission, Congenital heart disease, Two or more comorbidities, Chronic lung disease (bronchopulmonary dysplasia, cystic fibrosis, etc.).

Observational studies in adults and children have shown that multidisciplinary protocols, including caregiver education and surveillance guidelines for tracheostomy care, are associated with fewer complications.^26^^,^^27^ Based on the clinical characteristics and outcomes of tracheostomised patients in this case series, a multidisciplinary team (pediatric intensivists, surgeons, otolaryngologists, and palliative care specialists) developed a framework to support clinical decision-making, identify indications for pediatric palliative care, and guide airway management in ICU patients (Figure 1). This framework encompasses neonates with congenital or genetic disorders, infants younger than one year of age, malnourished infants, those with neurological impairments, patients presenting with a low functional status at admission, individuals with congenital heart disease, those with two or more comorbidities, and patients with chronic lung disease (including bronchopulmonary dysplasia, cystic fibrosis and related conditions).

All tracheostomized children included in this study met the World Health Organization criteria for palliative care, although only a small proportion were formally evaluated. Early and systematic improved assessment by a multidisciplinary pediatric palliative care team is essential, as it allows for individualized therapeutic planning, continuous alignment of expectations with families, and quality of care throughout the disease trajectory.

A clinical decision-making framework can serve as a practical tool to guide the timely initiation of palliative care and assist in ICU airway planning. Further studies are required to validate this framework within different healthcare contexts, as well as important qualitative research to explore family involvement in clinical decisions and post-discharge quality of life.

## Funding/support

This research received no specific grant from any funding agency in the public, commercial, or not-for-profit sectors.

## Data availability statement

The data that support the findings of this study are available from the corresponding author.

## Conflicts of interest

The authors declare no conflicts of interest.
